# Mycophenolic Acid-loaded Naïve Macrophage-derived Extracellular Vesicles Rescue Cardiac Myoblast after Inflammatory Injury

**DOI:** 10.1021/acsabm.3c00475

**Published:** 2023-09-29

**Authors:** Han Gao, Shiqi Wang, Zehua Liu, Jouni T. Hirvonen, Hélder A. Santos

**Affiliations:** † Department of Biomedical Engineering, W.J. Kolff Institute for Biomedical Engineering and Materials Science, University Medical Center Groningen, University of Groningen, Ant. Deusinglaan 1, 9713 AV Groningen, The Netherlands; ‡ Drug Research Program, Division of Pharmaceutical Chemistry and Technology, Faculty of Pharmacy, University of Helsinki, FI-00014 Helsinki, Finland

**Keywords:** exosomes, drug loading, macrophages, anti-inflammation, immunoregulation

## Abstract

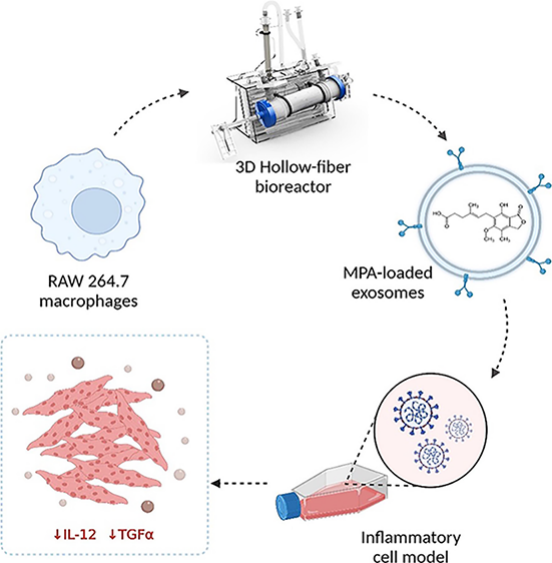

Exosomes are natural endogenous extracellular vesicles with phospholipid-based bilayer membrane structures. Due to their unique protein-decorated membrane properties, exosomes have been regarded as promising drug carriers to deliver small molecules and genes. A number of approaches have been developed for exosome-based drug loading. However, the drug loading capability of exosomes is inconsistent, and the effects of loading methods on the therapeutic efficacy have not been investigated in detail. Herein, we developed anti-inflammatory drug-loaded exosomes as an immunomodulatory nanoplatform. Naïve macrophage-derived exosomes (Mϕ-EVs) were loaded with the anti-inflammatory drug mycophenolic acid (MPA) by three major loading methods. Loading into exosomes significantly enhanced anti-inflammatory and antioxidation effects of MPA in vitro compared to free drugs. These findings provide a scientific basis for developing naïve macrophage-secreted exosomes as drug carriers for immunotherapy.

## 1 Introduction

Smart nanocarriers for drug delivery have been substantially exploited in recent decades.^1^ Currently, several synthetic delivery platforms are undergoing/leading to clinical translation, such as lipid-formulated nanoparticles.^2^ In addition to these synthetic biomaterials, extracellular vesicle-based drug carriers showed considerable prospects as a next-generation drug delivery system.^1^ Extracellular vesicles are lipid-bound nanoscale carriers secreted by almost all cell types. These vesicles contain biological contents derived from the parent cells, i.e., proteins, nucleic acids, and membrane molecules, which aid them to transfer to recipient cells for intercellular communication.^3^ In addition, extracellular vesicles have a distinct function in the maintenance of tissue homeostasis and biological regulation.^3^ In terms of biogenesis, they have different subclass nanosized particles, including microvesicles (50–1000 nm), exosomes (30–150 nm), and apoptotic bodies (>500 nm).^4^

Exosomes are a heterogeneous subset of extracellular vesicles formed by the fusion of multivesicular endosomes with the plasma membrane.^5^ Compared with microvesicles, exosomes have higher cholesterol content to maintain the membrane structure.^5^ As a major type of extracellular vesicles, exosomes contain various functional proteins and RNAs and can transfer them to target cells as intracellular mediators.^4^ Currently, several groups have demonstrated the important role of exosomes involved in different complications, particularly in the context of immune regulation and cancer therapy.^3,6^ For example, mesenchymal stem cell-derived exosomes (MSC-EVs) were shown to promote angiogenesis by activating anti-inflammatory responses via M2 polarization, which have great therapeutic potential for tissue regeneration.^7^

Due to their unique properties and natural sizes, exosomes are emerging as promising candidates for drug delivery. As an inherent carrier platform, exosomes hold unique properties for exogenous drug loading, including less immunogenicity, biocompatibility, and cell/tissue-specific orientation.^8^ Recent studies have demonstrated that exosomes can carry different types of drugs, including small molecular drugs, therapeutic genes, and biological cargo, such as peptides and proteins.^9^ However, the approaches for incorporating cargos into exosomes varied among previous studies, which mainly include (1) preloading before exosomes isolation, (2) passive drug loading, and (3) active drug loading.^9^ For small molecular drugs, the passive loading approach has been widely adopted, including the coincubation of drug with exosomes or with donor cells.^10^ For example, paclitaxel loaded in exosomes by passive diffusion, which mainly relies on the hydrophobic interaction and diffusion between the drug molecules and the lipid layer of the exosomes, significantly improves the antitumor effect with a loading efficiency of 9.2%.^11^ In contrast, macromolecular drugs were mostly loaded by actively loading, via electroporation,^12^ sonication,^13^ extrusion,^14^ or membrane permeability approach.^10^ Despite the individual reports about the successful loading of different drugs, limited attention has been dedicated to comparing loading methods and the subsequent effects on final therapeutic efficacy.

Herein, we demonstrated the feasibility of naïve macrophage-derived exosomes (Mϕ-EVs) as vehicle for incorporating the small molecular drug mycophenolic acid (MPA). In particular, we for the first time compared the encapsulation efficacy into Mϕ-EVs by using three commonly used drug-loading strategies. As a proof of concept, we further evaluated the therapeutic effects of Mϕ-EVs mediated nanosystem in a cardiac inflammatory cell model. Overall, the current study provides insight into the development of naïve macrophages-derived exosomes for drug delivery, which may present a useful therapeutic approach for cardiovascular diseases.

## 2 Experimental Section

### 2.1 Materials

DMEM high glucose, fetal bovine serum (FBS), and PBS (pH 7.4, 10× ) were purchased from ThermoFisher Scientific (Waltham, Massachusetts, USA). The hollow fiber bioreactor system was bought from KDBIO (Berstett, France), with the use of 20 kDa cutoff hydrophilic fibers (C2011) and a chemically defined protein-free serum CDM-HD. Human albumin (BSA), calcein AM, Dio dye, 2′,7′-Dichlorodihydrofluorescein diacetate (H2 DCFDA) and propidium iodide (PI) were purchased from Sigma-Aldrich (St. Louis, MO, USA). The prestained protein ladder (PL00001), primary, and secondary antibodies for Western blotting analysis were obtained from Proteintech (Cranbury, USA). The mycophenolic acid (MPA) drug was purchased from TCI (Basel, Switzerland). Enzyme-Linked Immunosorbent Assay of cytokines TNF-α, IL-4, and IL-12 were performed by using ABTS ELISA kits (Peprotech, Cranbury, USA) according to the manufacturer’s instructions.

### 2.2 Cell Culture

RAW 264.7 murine macrophage cells and H9C2 rat cardiac myoblast cells were purchased from the American Type Culture Collection (ATCC). All the cells were cultured in DMEM high glucose medium, supplemented with 10% fetal bovine serum and 1% penicillin (100 units/mL) and streptomycin (100 μg/mL), which were maintained in a humidified 5% CO_2_ incubator at 37 °C. To construct an in vitro inflammation cell model, H9C2 cells were seeded and cultured overnight for attachment, then treated with lipopolysaccharide (LPS) (10 μg/mL) for 48 h for stimulation.

### 2.3 Exosome Preparation and Characterization

RAW 264.7 Mϕs were grown in T75 flasks to obtain 100 million cells before being put into the hollow fiber 3D bioreactor. The hollow fiber bioreactor was used according to the manufacturer’s instruction. Briefly, the harvested medium was collected and centrifuged sequentially at 500 × *g* for 10 min, 2000 × *g* for 10 min, and 10,000 × *g* for 30 min, followed by filtering through 0.2 μm membrane filters to remove large particles. The exosomes derived from the standard 2D cell culture method or the hollow fiber 3D bioreactor were isolated and purified by the common sequential ultracentrifugation method.^15^ The exosome pellets were resuspended in 1 mL of PBS and washed again to remove the impurities. The *z*-average diameter and polydispersity index (PDI) of exosomes was characterized by dynamic light scattering (DLS), and the particle concentration of exosomes was quantified by nanoparticles tracking analysis (NTA). For transmission electron microscopy (TEM) analysis, isolated exosomes were loaded onto a Formvar coated copper grid and stained with 2% of phosphotungstic acid (PTA), followed by characterizing using a Talos F200i transmission electron microscope (TEM, ThermoFisher, USA). To evaluate the membrane integrity of Mϕ-EVs, calcein AM (1 mM in DMSO) was diluted in PBS to a final concentration of 10 μM, followed by resuspending exosomes in 100 μL working solution.^16^ The mixture was incubated at 37 °C for 20 min and then washed with PBS to remove free calcein AM/EVs. The positive events were detected by a NovoCyte flow cytometer (Agilent, USA). The stability of the EVs was evaluated for up to 3 weeks. Briefly, isolated EVs diluted in PBS supplemented with human albumin and trehalose (PBS-HAT) was stored at 4 °C.^17^ The size and dispersity of diluted EVs were detected by DLS at different time points (day 1, day 7, day 14, and day 21).

### 2.4 Drug Loading of Exosomes

EVs isolated from naïve macrophages were used for drug loading. Typically, vesicles suspended in PBS were mixed with drug in conditioned buffer (Tris-HCL, pH 7.4), which is constant in all of the drug loading experiments. For passive loading, EVs and the drug were incubated at 37 °C for different time points. For active loading, EVs and drug were incubated in 0.01% TritonX-100 at 37 °C for 10 min. The RAW264.7 macrophages were preincubated with 50 μg/mL of MPA to obtain donor cell-secreted drug loaded exosomes. All drug-loaded EVs were purified by PBS via the traditional ultracentrifugation method.^18^ The loaded amounts of small molecular drug MPA was quantified by HPLC, with the method adopted from the previous study.^19^

### 2.5 Cellular Uptake and Biocompatibility

The cell viability of exosomes was determined by the CellTiter-Glo Luminescent Cell Viability Assay. Briefly, cells were seeded in a 96-well plate at 1 × 10^4^ cells per well, followed by incubation overnight for attachment. Afterward, cells were treated with different concentrations of exosomes for another 48 h, and the cell viability was measured according to the manufacturer’s instructions. To assess the cellular uptake of exosomes on H9C2 cells, the exosomes were stained with Dio dye at a final concentration of 1 μM, followed by dialyzing in a microdialysis plate (ThermoFisher, USA) to remove the free dye. The Dio-labeled exosomes was then incubated with cells for another 4 h, followed by analyzing by flow cytometry analysis or confocal assay.

### 2.6 Therapeutic Evaluation of MPA-Loaded Exosomes

In the cell death mechanism studies, the cells were pretreated with different groups for 48 h. For bare MPA treatment, the MPA drug at 50 μg/mL was applied as a comparable negative group. Afterward, cells were detached and washed by PBS, stained with PI at 1 μg/mL, and resuspended in 0.3 mL of PBS. Visible positive cell events were quantified by a NovoCyte flow cytometer (Agilent, USA). The cellular ROS level was visualized by a DCFDA assay. Briefly, cells (pretreated with different EVs nanoformulations) were washed and stained with DCFDA for 30 min, followed by analysis with a fluorescent microscope (Leica, Germany). The expression levels of inflammatory cytokines were determined by an ELISA assay. A sandwich ELISA system was constructed according to the manufacturer’s instructions. The prepared samples were incubated with detection antibodies for 2 h, followed by washing and incubation with secondary antibodies for another 1 h. After the ABTS buffer was added, the plate was measured at 416 nm by a BioTek Fluorescence Microplate Reader (Lonza, Switzerland).

### 2.7 Statistical Analysis

Data analysis was performed using GraphPad Prism 9.0. The significance of the results was determined by One-way ANOVA analysis or two-tailed Student’s *t* test. *p* < 0.05 was defined as statistically significant. *p < 0.05; **p < 0.01; ***p < 0.001; ****p < 0.0001; ns, no statistical significance.

## 3 Results and Discussion

### 3.1 Preparation and Characterization of Naïve Mϕ-Derived Exosomes

**Figure 1:**
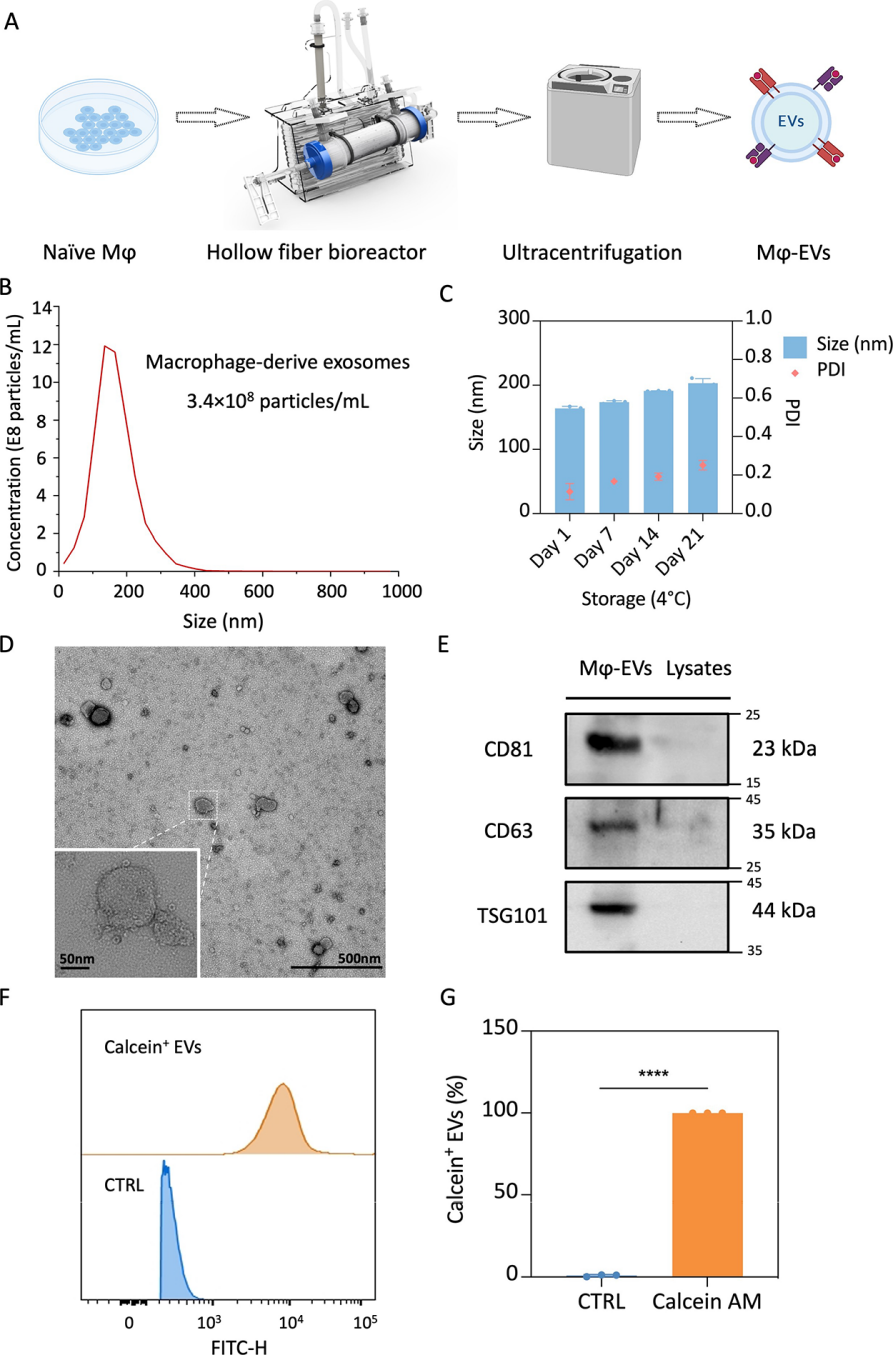
Preparation and characterization of naïve macrophage-derived exosomes. (A) Schematic illustration of a 3D hollow fiber bioreactor-based exosomes preparation system. (B) Size distribution of Mϕ-EVs quantified by NTA analysis. (C) Long-term storage stability of Mϕ-EVs in conditioned buffer. (D) Morphology of Mϕ-EVs was determined by TEM analysis. (E) Western blotting assay was performed to detect the biomarkers of Mϕ-EVs. (F) and (G) Calcein-AM based flow cytometry analysis was performed to investigate the membrane integrity of Mϕ-EVs. Two-tailed Student’s *t*-test was used to compare the differences. ****, *p* < 0.0001. Created with BioRender.com.

Herein, we chose naïve macrophage as the donor cell to produce exosomes because of its potential immunoregulatory roles and the ability to escape immunological surveillance.^20^ To obtain large quantities of naïve Mϕ-EVs, in the current study, we applied a hollow-fiber bioreactor 3D system for a continuous production of biological products (Figure 1A). We chose this bioreactor system based on the following considerations: (1) a flow-based culture system could provide necessary environment to maintain cell viability and homeostasis, which is the prerequisite for subsequent EVs preparations;^21^ (2) taking advantages of this well-controlled cartridge system, cells can be expanded fast that allows for large-scale production of conditioned medium without contamination;^22^ and (3) from an industrial perspective, continuous production of high-quantify EVs has potential translation value for clinical application.^18,21^ Herein, we found that macrophages seeded in the hollow-fiber system corresponded to a 10-fold linear increase in EVs amounts compared to the traditional 2D method (Figure S1).

The isolated Mϕ-EVs showed a size distribution in a narrow range, as analyzed by nanoparticle tracking analysis (NTA), suggesting the homogeneity of the exosome populations (Figure 1B). Next, we checked the storage stability of Mϕ-EVs in the preconditioned buffer. As shown in Figure 1C, EVs can retain their size and monodispersity for at least 14 days when stored at 4 °C, which was also confirmed in serum conditions for 7 days’ storage. Characterized by DLS analysis, this EVs-based nanosystem presented good stability in serum (Figure S2). Such excellent colloidal stability is a prerequisite to preserve the EVs compositions and facilitate the clinical translation of EVs-based nanoformulations.^17^ We then performed TEM analysis to check the morphology of Mϕ-EVs. As depicted in Figure 1D, a typical lipid-enclosed membrane structure was observed, and the particle size corresponds well with NTA and DLS results.

Moreover, the identification of exosome membrane is critical for preserving biological functions and transferring cargos as the drug delivery system.^8^ Different from bioengineered liposomes, the compositions of exosome membranes are mainly consisted of proteins and lipids, which contribute to their stability, surface charge, and transmembrane capability.^23^ Therefore, in the current study, we detected the exosome-specific biomarkers by Western blotting. As shown in Figure 1E, three typical exosomal markers, including CD81, CD63 and TSG101, were enriched in EVs samples, whereas low-to-no expression in the lytic samples of the supernatant was observed. We further detected a cell-associated protein, calnexin, to clarify the purity of naïve macrophages-derived EVs. As shown in Figure S3, calnexin was observed in cell lysates in a dose-dependent manner, whereas no expression was observed in exosomes. These results indicated that the exosomes isolated from naïve macrophages cultured in the 3D bioreactor system have desirable physiochemical properties, storage stability, and membrane integrity as nanocarriers for drug delivery applications.

In addition to the physiochemical properties characterizations, we also evaluated the membrane integrity of exosomes isolated, as it is crucial for their bioactivities and potential applications as delivery agents.^5^ To achieve this, we adopted a calcein-acetoxymethyl (AM)-based strategy to quantify the intact EVs.^16^ First, we incubated the isolated EVs with 10 μM calcein AM, which is nonfluorescent and membrane-permeable. Upon hydrolysis of the acetoxymethyl ester moieties by esterase inside exosomes, the fluorescent carboxyfluorescein is relatively membrane impermeant, thus retaining in EVs. If the EV membrane is compromised, carboxyfluorescein will leak from the EVs, with diminished fluorescence. Therefore, by analyzing the positive events after calcein AM labeling, it is possible to identify intact EVs. The positive events for EVs labeling were analyzed by flow cytometry, as evident from Figure 1F,G, nonpermeabilized EVs were positive for calcein AM labeling, with a fluorescent population at 99.9%. These data suggested the exosomes isolated from naïve macrophages were intact biological vesicles, which is the preliminary condition for the EV-based drug delivery system.^24^

### 3.2 Biocompatibility and Cellular Uptake of Naïve Mϕ-Derived Exosomes

**Figure 2:**
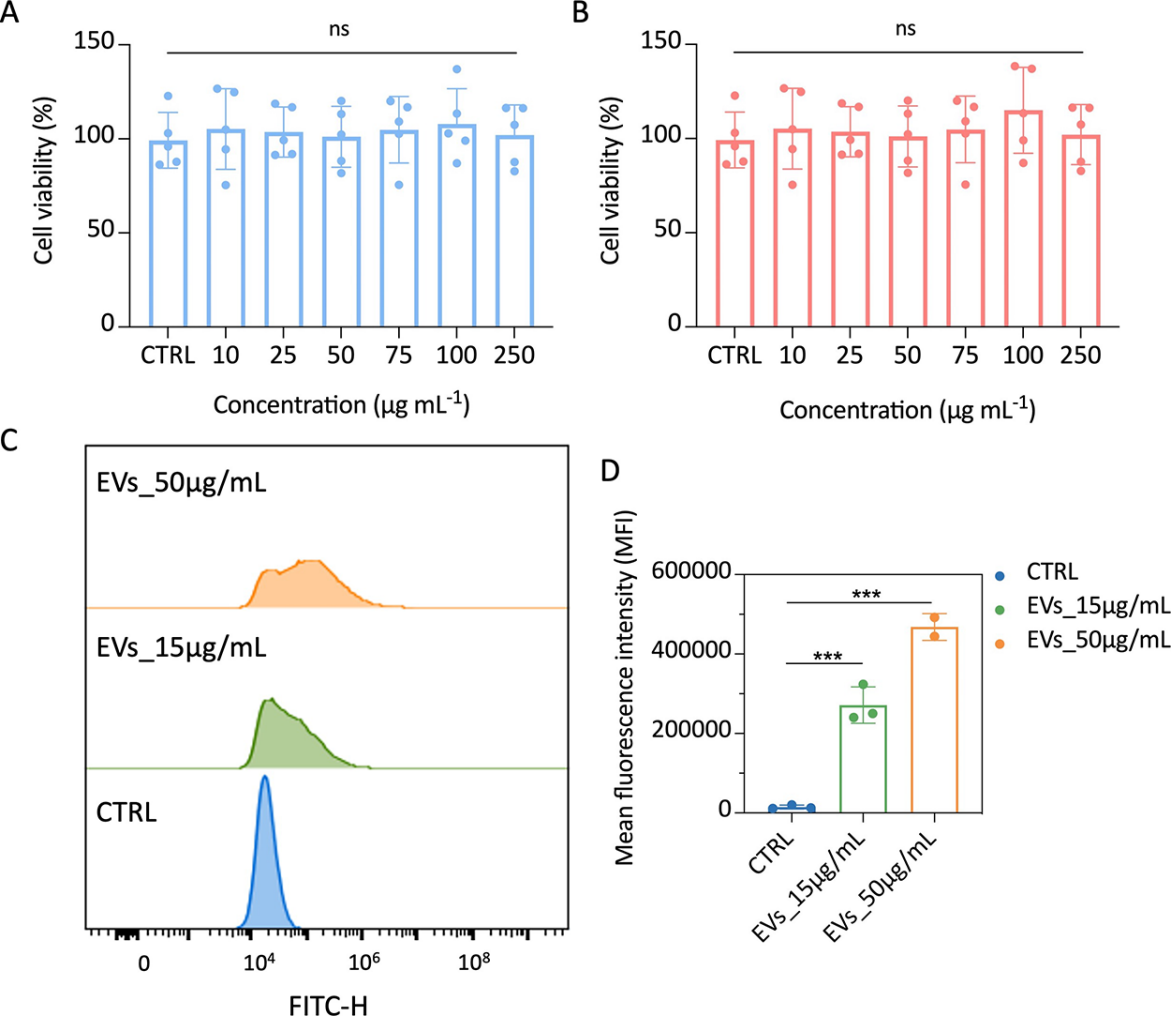
Biocompatibility and cellular uptake of naïve macrophage-derived exosomes. The cytotoxicity of Mϕ-EVs was evaluated on H9C2 cells (A) and RAW 264.7 cells (B). (C) and (D) Flow cytometry analysis was performed to evaluate the cellular uptake of Mϕ-EVs on H9C2 cells. (μg/mL: EVs’ concentration). The significance of the results was determined by One-way ANOVA analysis and two-tailed Student’s *t*-test. ***, *p* < 0.001. ns, no significance.

Before drug loading and delivery, we first investigated the cytotoxicity of Mϕ-EVs against murine cardiac myoblast (H9C2) and macrophage (RAW 264.7) cell lines. The CellTiter assay showed that the viability of these cells was not affected by exposure to Mϕ-EVs for 48 h, up to the highest concentration at 250 μg/mL (Figure 2A,B). These results are consistent with the reported studies, showing that EVs have inherent features desirable for an ideal drug delivery system, including good biocompatibility for potential clinical translation.^25^

Having demonstrated the biocompatibility of Mϕ-EVs, we investigated the cellular uptake on H9C2 cells, which will be used for further therapeutic tests. Naïve exosomes were labeled with the lipophilic dye Dio and incubated with H9C2 cells for 4 h, followed by fluorescence detection via flow cytometry analysis. As evident from Figure 2C,D, the uptake percentage of Mϕ-EVs in H9C2 cells increased in a concentration-dependent manner, reaching 43.6% at the concentration of 50 μg/mL. To further evaluate the uptake of Mϕ-EVs on H9C2 cells, Dio-labeled EVs were detected by fluorescence confocal microscopy after coincubation. Consistent with the flow cytometry results, considerable cellular uptake was observed when cells were incubated with varying concentrations of Mϕ-EVs. By increasing the concentration, more Mϕ-EVs were shown to accumulate in the cytoplasm (Figure S4). These results confirmed the intrinsic property of exosomes for cellular internalization, which may depend on the endocytosis pathways for EVs derived from immune cells.^26^

### 3.3 Mϕ-Exosomes as a Natural Drug Delivery System

**Figure 3:**
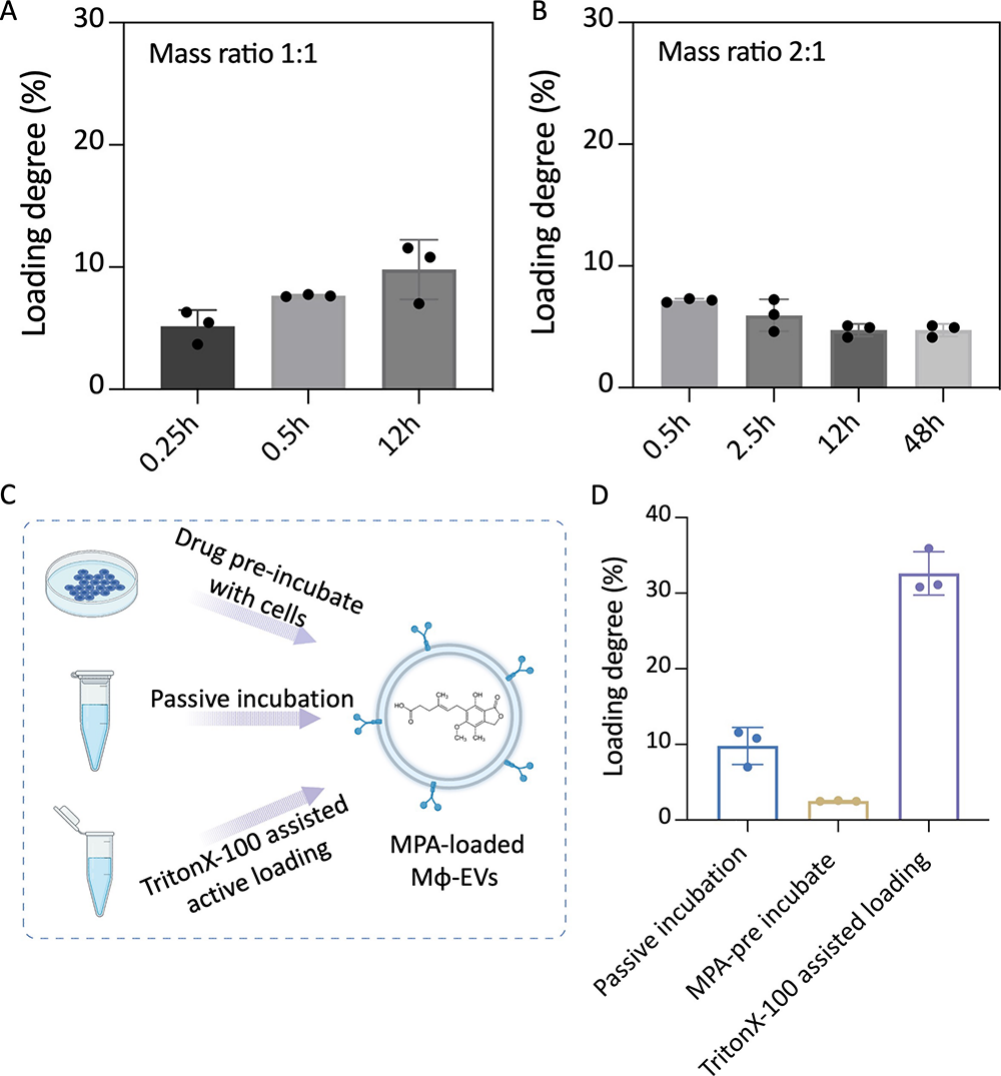
Mϕ-EVs as a natural drug delivery system. Drug loading efficacy determined by HPLC with the mass ratio between EVs and drug at (A) 1:1 and (B) 2:1. (C) Schematic illustration for MPA-encapsulated Mϕ-EVs with three major drug loading methods. (D) Drug loading degree based on different methods was quantified by HPLC. Created with BioRender.com.

As a result of the unique properties of these small, lipid-bound nanoparticles, EVs are also being explored for the delivery of therapeutics as nanocarriers, in particular, the small molecule drugs.^1^ However, the drug loading capacity of EVs as carriers was not investigated in detail.^27^ Previous studies suggested that the loading efficiency into EVs may be different due to exosomes origins, drug properties, and loading methods.^28^ However, a few studies reported on the use of naïve macrophage-derived exosomes for incorporating drug of interest, combining the application on cardiac systems. Aligning toward these issues, we first sought to investigate the drug-loading properties of Mϕ-EVs. We selected mycophenolic acid (MPA) as a model drug due to its hydrophobicity, which allows for incorporation into EVs’ membranes^24^ and its anti-inflammation therapeutic effects.^29^ First, the incorporation of MPA in Mϕ-EVs was performed by passive incubation, which is the most commonly used method for EV drug loading. The drug loading efficacy was determined by the high-performance liquid chromatography technique (HPLC). As shown in Figure 3A, with the mass ratio (w/w) between EVs and drug at 1:1, the loading degree (LD) of MPA increased with incubation time and reached 9.8 ± 2.4 (wt %) after 12h incubation at 37 °C. Moreover, we also increased the amounts of Mϕ-EVs to check the influence on the loading efficiency. However, there was a negligible difference regarding the loading degree of MPA at 2:1 (w/w) compared with the ratio 1:1 (Figure 3B). This may be due to the fact that at 1:1 w/w ratio, the EV membranes were already saturated with MPA.^30^ Therefore, we chose the passive loading condition at w/w 1:1, 12 h at 37 °C for further analysis.

Next, to further assess whether other loading methods could improve the loading capacity of EVs, we have examined LD by coincubation MPA and EVs with 0.01% of Triton X-100 or by preincubation MPA with secreting cells. Both are also common strategies for the encapsulation of exogenous EVs cargos,^30^ as illustrated in Figure 3C. As shown in Figure 3D, when the cells were preincubated with MPA drugs, the loading efficiency was the lowest (2.5 ± 0.1 wt %). In contrast, the loading efficiency was significantly improved with the addition of 0.01% of TritionX-100. A much higher loading efficiency was achieved at 32.6 ± 2.9 wt % in comparison to other methods. We further investigated the influence on membrane integrity of exosomes by treating them with 0.01% of TritionX-100. The prepared EVs pellets were subjected to resuspend in 0.01% of Triton X-100, followed by incubating in 10 μM calcein AM for 20 min and washing with PBS. As shown in Figure S5, there was negligible difference on EV-labeled positive events between the control group and the Triton X-100 pretreated group, suggesting the vesicle membrane was only temporarily permeabilized during drug loading and able to reseal after the removal of Triton X-100.^31^

The development of the EV-based drug delivery platform was regarded as a next-generation therapeutic strategy.^1^ However, in-depth characterization of the loading efficacy of small-molecule drugs into EVs remains inconsistent and unclear, which is mainly associated with the loading strategies and incorporated drug properties.^9^ Therefore, assessing drug-loaded properties in a specific EVs type is beneficial for addressing obstacles in this emerging field. According to a previous study, exosomes released from macrophages exhibited extraordinary ability regarding the interaction with target cells, suggesting the profound effects of macrophages-derived EVs as delivery vehicles.^13^ Moreover, in a recent study, the authors have compared the encapsulation efficiency of Dox-loaded EVs by using six different strategies.^32^ As a result, the encapsulation ratios were varied depends on the loading strategies, among which surfactant-treatment showed superior loading efficiency compared to other physical approaches, such as extrusion and sonication.^32^ Inspired by these studies, herein, we adopted naïve macrophages-derived exosomes as a drug delivery platform and comprehensively compared the loading capacities by using three commonly used strategies. In consistence with previous studies, we found that the active loading method contributed to enhance the hydrophobic-drug loading into EVs. From a clinical perspective, this study is expected to facilitate the development of EVs-based cell-free therapies.

### 3.4 Anti-Inflammation Effects of MPA-loaded Mϕ-EVs Nanoplatform

**Figure 4:**
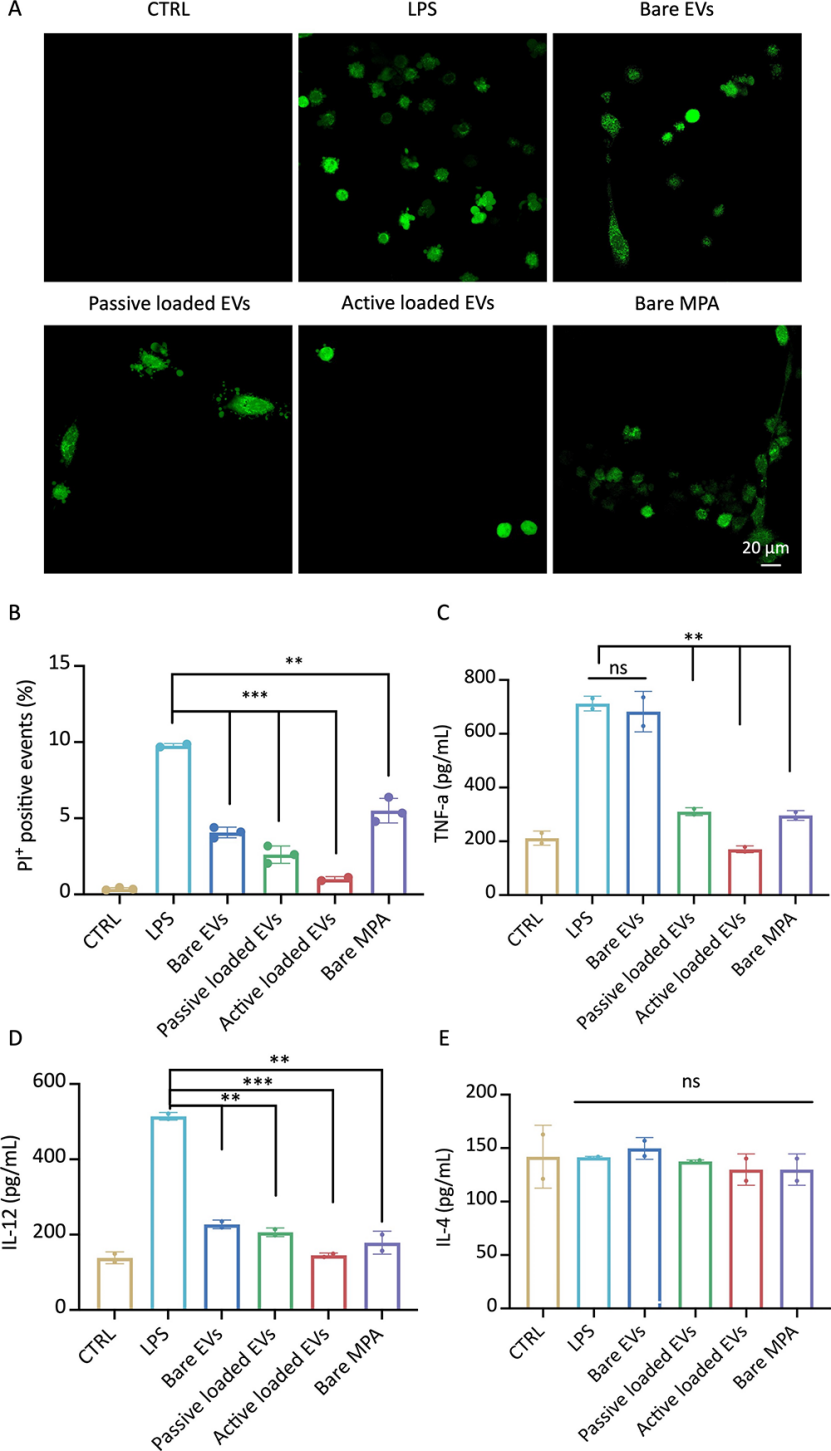
MPA-loaded Mϕ-EVs nanoplatform for inflammatory therapy. (A) DCFDA assay was performed to visualize the intracellular ROS level after treatment with different groups (Bare MPA: 50 μg/mL) (scale bar: 20 μm). (B) Percentage of PI-positive cell populations was detected by flow cytometry analysis. (C–E) Expression levels of cytokines were determined by the ELISA assay. The significance of the results was determined by One-way ANOVA analysis and two-tailed Student’s *t*-test. ns, no statistical significance; *p < 0.05, **p < 0.01, ***p < 0.001.

Having confirmed the drug-loading potential of Mϕ-EVs, the therapeutic effects of these exosomal vesicle-based nanoformulations were investigated in vitro. To achieve this, the rat embryonic heart-derived myogenic cell line H9C2 was stimulated by lipopolysaccharides (LPS) to induce a biomimetic inflammatory environment, which is a well-established cell model as reported previously.^33^ Mϕ-EVs (with or without MPA) were incubated with LPS-stimulated H9C2 cells for 48 h, followed by flow cytometry analysis to assess the cell viability after propidium iodide (PI) staining. As evident from Figure 4B, LPS stimulation increased the PI-positive cell percentage to 9.78%, suggesting inflammatory cell death. Bare exosomes showed a considerable therapeutic effect when incubated with H9C2 cells, which could be attributed to the immunomodulatory property of macrophage secreted exosomes.^20^ The free MPA group also showed a slight decrease in the PI-positive population, which could be attributed to the anti-inflammatory effects of MPA itself.^19^ Whereas enhanced cell viability was observed in cells treated with MPA-loaded EVs, the active-loading group assisted by Triton X-100 showed the best therapeutic efficacy, suggesting the antinecrotic effects of this exosomes-based nanomedicine.

Subsequently, we assessed whether the activated cell death program was associated with oxidative stress. For this, LPS-stimulated H9C2 cells were incubated at the same conditions as described above, and the intracellular level of reactive oxygen species (ROS) was characterized by oxidant-sensing fluorescent probe 2′,7′-dichlorodihydrofluorescein diacetate and imaged by fluorescence microscopy. As shown in Figure 4A, MPA-loaded exosomes (either passive or active loading method) showed a significantly lower level of ROS compared to LPS/free drug, which was further confirmed by quantitatively analysis (Figure S6). Levels of cellular ROS are modulated by the balance between the cellular processes that produce ROS and the processes that eliminate them.^34^ It has been proposed that the overgeneration of ROS may initiate cell death processes through inactivating dual specificity phosphatases.^34^ For example, pharmacological inhibition of JNK activity could significantly protect against necrosis induced by ROS and TNF.^35^ In our study, we found that the activated cell death was correlated with higher levels of ROS, which could be rescued by adding MPA-encapsulated EVs, suggesting the antioxidation effects of the Mϕ-EV nanoplatform.

Next, to assess whether Mϕ-EV-mediated drug delivery could have therapeutic effects in an inflammatory microenvironment, the cytokine production was measured after 48 h incubation. As shown in Figure 4C,D, MPA-loaded EVs inhibited inflammation by reducing the production of proinflammatory cytokines, tumor necrosis factor α (TNF-α), and interleukin 12 (IL-12), suggesting the successful delivery of MPA drugs to the H9C2 cells. TNF-α mediated p65-NF-kB signaling is essential for engaging cell-death mechanisms, especially necroptosis.^36^ In this study, we found that MPA-loaded Mϕ-EVs protect against H9C2 necroptosis by downregulating TNF-α. Interestingly, Triton X-100 assisted active drug loading showed a significantly lower expression profile of proinflammatory cytokines than those of other groups, which could be attributed to the high loading efficacy of Mϕ-EVs. Moreover, our findings revealed that naïve Mϕ-EVs alone cannot downregulate TNF-α expression compared with the control group, which is in agreement with previous studies,^37^ in which exosomes isolated from naïve bone-marrow-derived-macrophage (BMDM) was shown to have little effect on regulating TNF-α expression. Regarding the IL-12 level, bare EVs already exerted a significant therapeutic efficacy in downregulating the cytokine, which may result from the originate macrophages. An important characteristic of EVs associated with bioactivity is their inherent biological molecules reflective of their origin.^5^ Along with previous study, exosomes can exert significant protective effects even without any drug loaded, suggesting the potential combinatory effects by coincubation of EVs and drugs.^38^ Our data indicated that naïve macrophages-derived EVs exerted immune-privileged property, which might be beneficial for systematic delivery regarding prolonged retention time and evaded from the mononuclear phagocyte system. Indeed, the loading of MPA in Mϕ-EVs further enhanced the downregulation of IL-12, making it back to the baseline level (control group). Additionally, the anti-inflammatory cytokine IL-4 showed negligible changes after Mϕ-EV treatments (with/without drug loading), which could be attributed to the phenotypes of donor macrophages (Figure 4E).^20,39^

## 4 Conclusions

We isolated exosomes from RAW 264.7 macrophages and characterized their capacity as a naïve drug delivery platform, which was further evaluated in an inflammatory cell model. By comparing three main loading strategies, different drug loading degrees were achieved as quantified by HPLC, which provided fundamental insights into the explorations of drug delivery abilities of naïve macrophages-derive exosomes. Furthermore, the anti-inflammatory drug MPA loaded EVs successfully reduced the intracellular oxidative stress and proinflammatory cytokine levels, thus relieving necrotic cell death on H9C2 murine cardiac myoblasts. Taken altogether, Mϕ-EVs are promising nanocarriers for encapsulating small molecule drugs and have great potential for further development in anti-inflammatory therapies.

## Supporting Information Available

Comparison of yield of exosomes extracted by conventional method and hollow fiber bioreactor-based approach; storage stability of Mφ-EVs in serum; quantitative analysis of calnexin protein in cell lysates and exosomes; cellular uptake of Dio-labeled EVs was evaluated by confocal assay; membrane integrity of EVs was evaluated via calcein-AM based method; and comparison of fluorescence intensity among different groups in DCFDA assay (PDF)
