# Supplementary material for: Mycophenolic Acid-loaded Naïve Macrophage-derived Extracellular Vesicles Rescue Cardiac Myoblast after Inflammatory Injury
Source: ACS Appl Bio Mater. 2023 Sep 29;6(10):4269–76. doi: 10.1021/acsabm.3c00475 (PMC10583195; doi:10.1021/acsabm.3c00475)
Supplement: Supplementary file 1 — mt3c00475_si_001.pdf [file mt3c00475_si_001.pdf]

Supporting Information

Mycophenolic acid-loaded naïve macrophage-derived extracellular vesicles rescue cardiac myoblast after inflammatory injury

Han Gao[1, 2], Shiqi Wang[2,*], Zehua Liu[2], Jouni T. Hirvonen[2], Hélder A. Santos[1, 2,*]

1. Department of Biomedical Engineering, W.J. Kolff Institute for Biomedical Engineering and Materials Science, University Medical Center Groningen, University of Groningen, Ant. Deusinglaan 1, 9713 AV Groningen, The Netherlands

2. Drug Research Program, Division of Pharmaceutical Chemistry and Technology, Faculty of Pharmacy, University of Helsinki, FI-00014 Helsinki, Finland

*Corresponding authors: shiqi.wang@helsinki.fi; h.a.santos@umcg.nl

Supplementary Figures

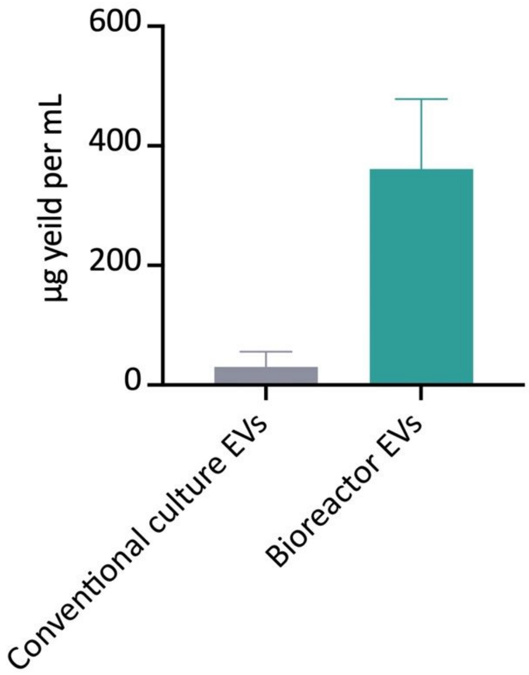

Figure S1. Comparison of yield of exosomes extracted by conventional method and hollow fiber bioreactor-based approach. The concentration of exosomes was quantified by bicinchoninic acid (BCA) protein assay (unit µg/mL).

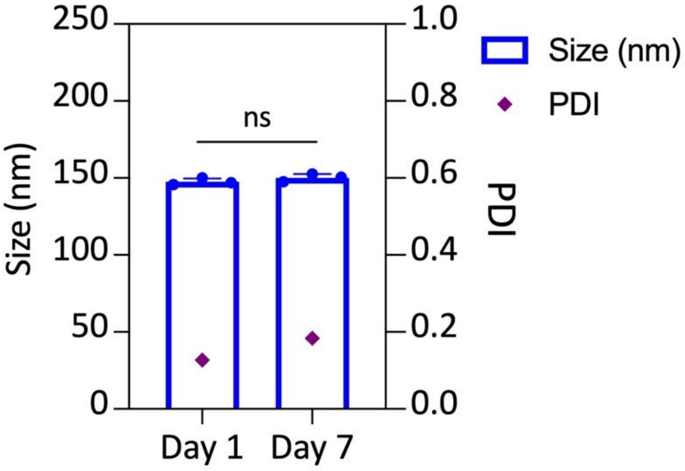

Figure S2. Storage stability of Mϕ-EVs in serum. Dynamic light scattering analysis was adopted to determine the size distribution and polydispersity (PDI) of macrophage-derived exosomes. ns: no statistical significance.

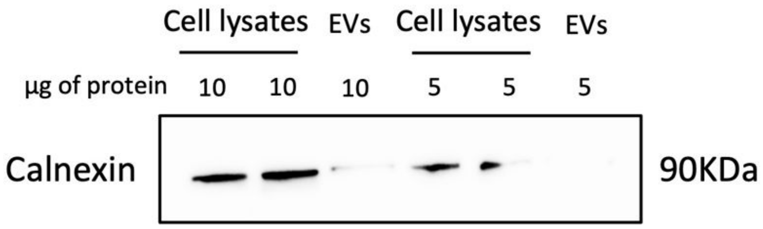

Figure S3. Quantitative analysis of Calnexin protein in cell lysates and exosomes. The expression levels of calnexin were detected by western blotting.

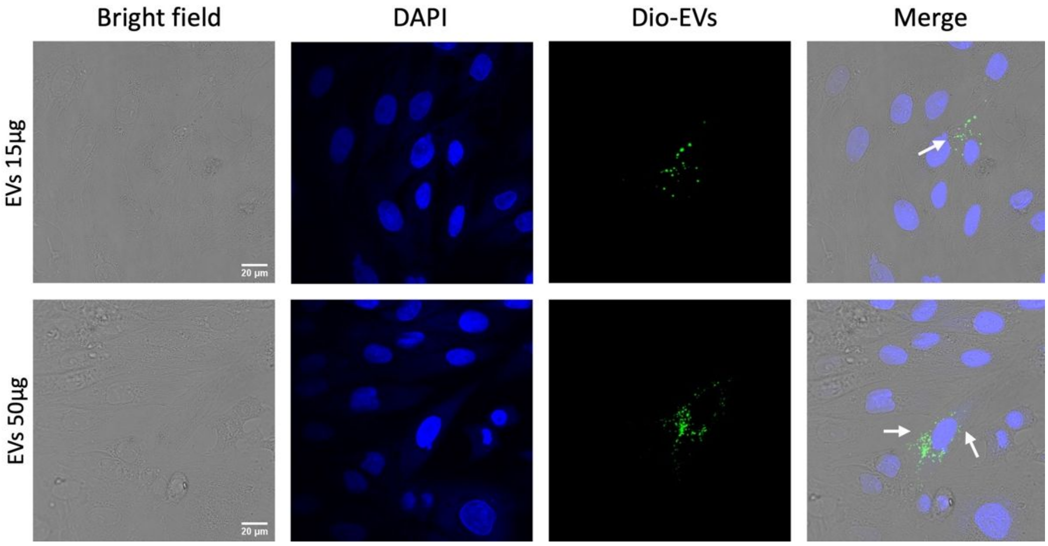

Figure S4. Cellular uptake of Dio-labelled EVs was evaluated by confocal assay. Scale bar: 20 μm.

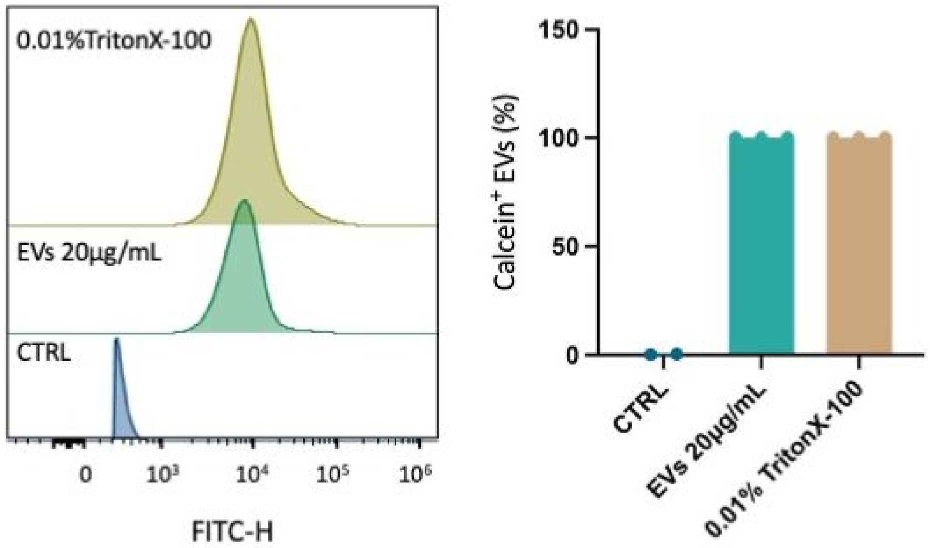

Figure S5. Membrane integrity of EVs was evaluated via calcein-AM based method (0.01% TritonX-100: EVs were pre-treated with TritonX-100 to temporarily permeabilize the membrane).

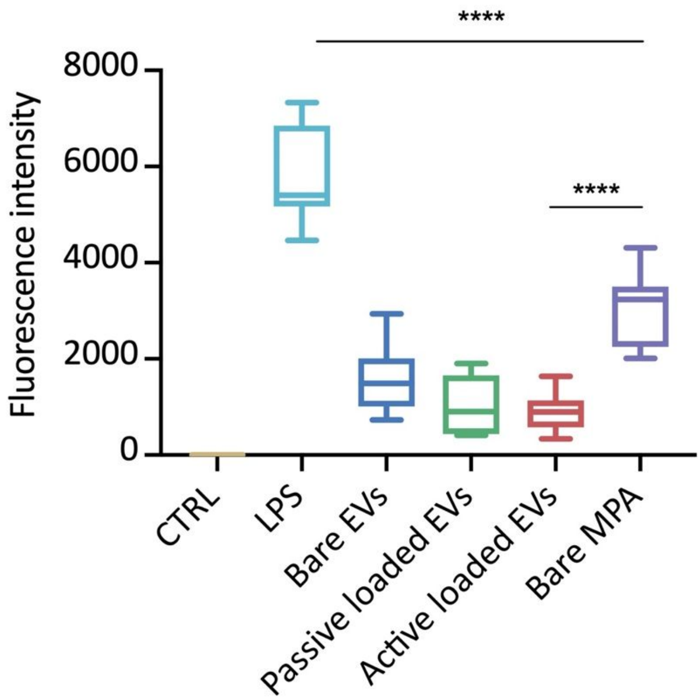

Figure S6. Comparison of fluorescence intensity among different groups in DCFDA assay. ****, p<0.0001.
